# Urinary Retention in a Patient With an Artificial Urinary Sphincter: A Case Report of a Rare Cause of Urinary Retention

**DOI:** 10.7759/cureus.11259

**Published:** 2020-10-30

**Authors:** Joseph Anthony Demirjian, Elaine H Situ-LaCasse

**Affiliations:** 1 Emergency Medicine, University of Arizona College of Medicine - Tucson, Tucson, USA; 2 Emergency Medicine, University of Arizona, Tucson, USA

**Keywords:** point-of-care-ultrasound, ultrasound, bedside ultrasound, emergency medicine, urology, urinary retention, artificial urinary sphincter, ams800

## Abstract

Urinary retention is the inability to spontaneously void with lower abdominal or suprapubic pain caused by infection, trauma, obstruction, medications, or neurological etiologies. Acute urinary retention (AUR) is a urological emergency often seen in males presenting to the emergency department (ED). AUR is frequently seen in men over the age of 60 and approximately one-third of men over the age of 80. A 61-year-old Spanish-speaking male, with a history of prostate cancer and prostatectomy with the recent insertion of an artificial urethral sphincter two months prior, presented to the ED with urinary retention, complaining of malfunction in his artificial sphincter with worsening abdominal pain, distention, urinary urgency, and nausea. A bladder scan demonstrated 450 ml of urine. Bedside ultrasound (US) showed moderate bilateral hydronephrosis and hydroureter. After consultation with urology, they revealed that the patient did not understand how to properly use his implanted device. Urology experts have recommended minimal urethral instrumentation in patients with artificial urinary sphincters due to the risk of complications. Although we present a rare cause of urinary retention, emergency physicians should avoid catheterization in these patients. Bedside renal ultrasound is useful for the diagnosis of hydronephrosis and hydroureter and confirmation of pump and balloon placement. We recommend a prompt urology consultation. This case is an important example of appropriate postoperative education and close-ended communication. Certified interpreters should be used to avoid communication barriers and complications.

## Introduction

Urinary retention is defined as the inability to spontaneously void associated with lower abdominal or suprapubic pain commonly caused by infection, trauma, obstruction, medications, or neurological etiologies [1]. Acute urinary retention (AUR) is a urological emergency most often seen in males presenting to the emergency department (ED). AUR is most frequently seen in men over the age of 60 and approximately a third of men over the age of 80 [2]. In contrast, AUR is rare in women, with a female-to-male incidence rate ratio of 1:13 [3-4]. Benign prostatic hyperplasia (BPH) is the predominating cause of AUR, but other causes that may be encountered in the emergency department include spinal cord injuries (eg, tumors, abscesses, or trauma), pharmacological etiologies (eg, opiates, sympathomimetics, or anticholinergics), acute prostatitis, and urethral trauma [5-9]. We present a rare cause of AUR seen in the emergency department.

## Case presentation

A 61-year-old, Spanish-speaking male, with a history of prostate cancer and prostatectomy, who underwent the recent insertion of an artificial urethral sphincter two months prior, presented to the emergency department with urinary retention. He was complaining of malfunction in his artificial sphincter associated with worsening abdominal pain, abdominal distention, urinary urgency, and nausea. The patient reported an inability to void in the past several days because the control for the artificial urinary sphincter was malfunctioning. The patient showed the physicians a small keychain with a control switch and stated that the switch no longer functioned. He was able to urinate in very small volumes, but he was uncomfortable with his distended bladder. He denied fever, hematuria, vomiting, diarrhea, or sick contacts.

**Figure 1:**
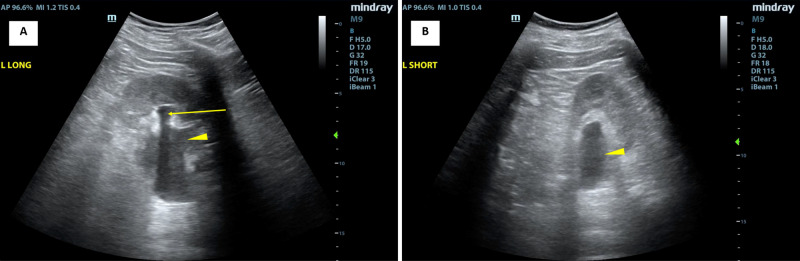
Left-sided renal ultrasound images The left-sided renal ultrasound image shown in the long axis view (A) demonstrates moderate hydronephrosis (marked by the arrow) and hydroureter (marked by the arrowhead). The short-axis view shows hydroureter.

**Figure 2:**
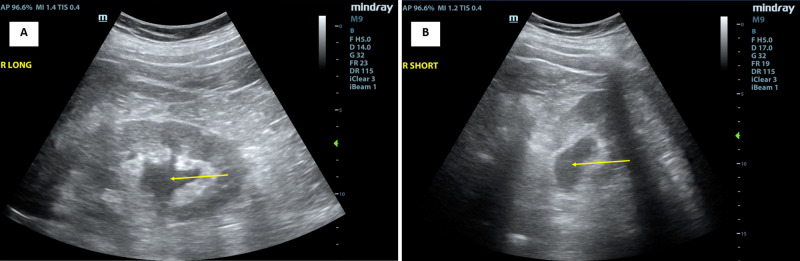
Right-sided renal ultrasound images Right-sided renal ultrasound images in the long-axis view (A) and the short-axis view (B) demonstrate moderate hydronephrosis (marked by arrows).

**Figure 3:**
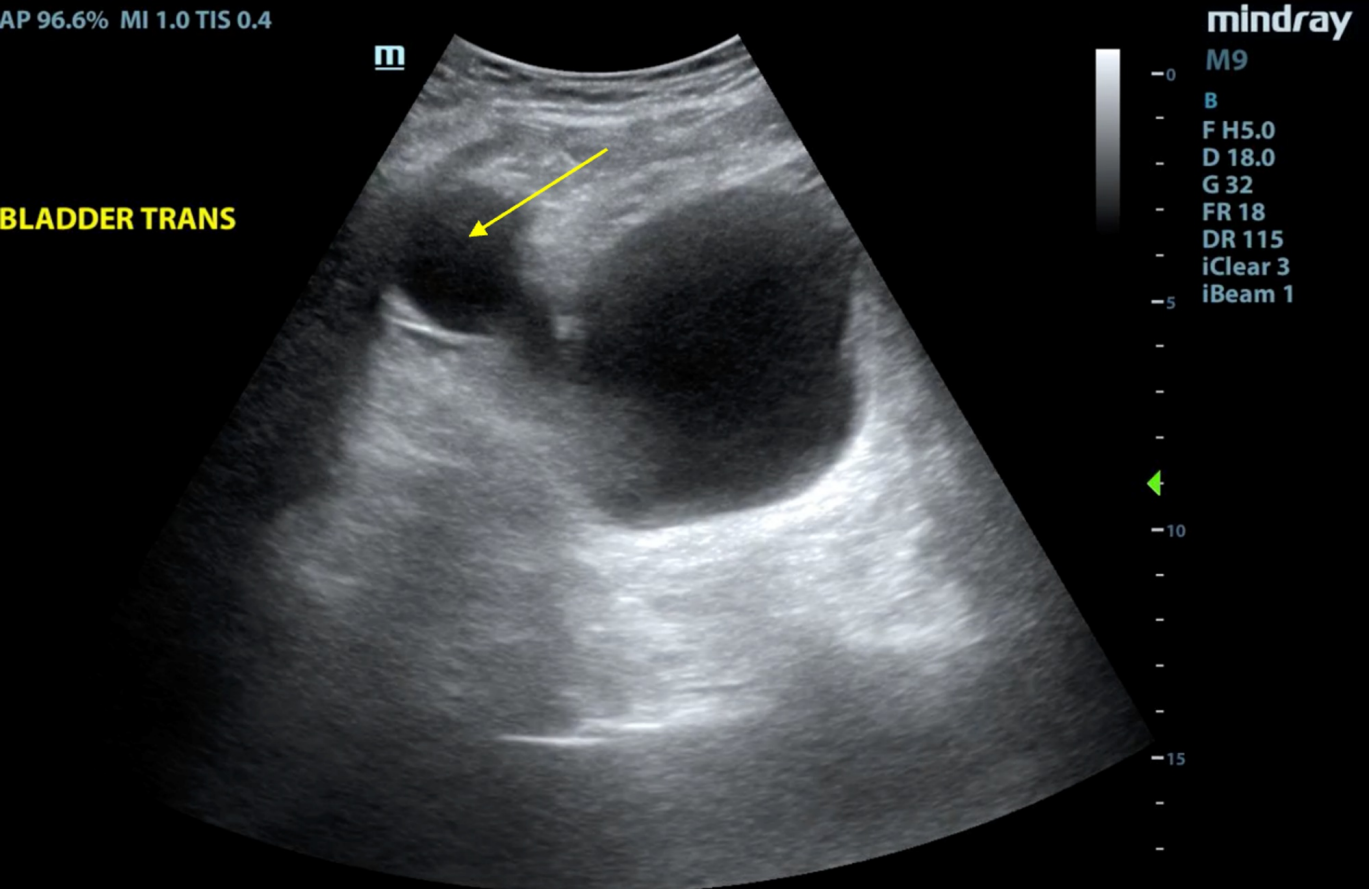
Bladder ultrasound image The bladder ultrasound image in the transverse view prior to voiding shows a right-sided “cystic mass” that is consistent with the normal positioning of the balloon of the artificial urinary sphincter at the right iliac fossa, with appropriate appearance (marked by the arrow).

Vitals were unremarkable. His exam was significant for mild abdominal distension and suprapubic tenderness without costovertebral angle tenderness. Labs were unremarkable without evidence of leukocytosis, electrolyte abnormalities, or renal function abnormalities. Urinalysis was also negative for blood or signs of infection. A bedside renal ultrasound revealed bilateral anechoic areas in the renal pelvis, which is consistent with bilateral hydronephrosis, and an anechoic area outside of the renal pelvis, which is consistent with hydroureter (Figures 1-2). Bedside ultrasound also revealed the appropriate positioning and appearance of the artificial urinary sphincter balloon (Figure 3). A bladder scan revealed approximately 450 ml of urine.

The urology team was consulted during the patient’s ED stay. Further history and exam revealed that the patient did not understand how to properly use his implanted device. The keychain that the patient had was a model used for education, and it did not control the sphincter. Urology consultants provided proper education on how to activate the artificial urinary sphincter in his right hemi-scrotum with a Spanish translator present. The patient demonstrated no significant post-void residual volumes (defined as <200 ml) on a repeat bladder scan.

The patient was discharged and scheduled to follow up with urology as an outpatient. A review of his records showed that he was able to demonstrate a proper understanding of how to use his device during his urology follow-up visit.

## Discussion

The diagnosis of AUR is made by demonstrating retained urine via bladder ultrasound or catheterization. Ultrasound serves as a useful non-invasive tool in the evaluation of patients with AUR. The common etiologies of urinary retention in the ED are often managed with bladder decompression and straight catheterization. Typically, a bladder volume of ≥300 ml on ultrasound has been suggested to confirm the diagnosis of urinary retention and warrant decompression [6].

We present a unique case of a patient who presented with a suspected malfunction of his artificial urinary sphincter, a rare cause of urinary obstruction of which emergency physicians should be aware.

**Figure 4:**
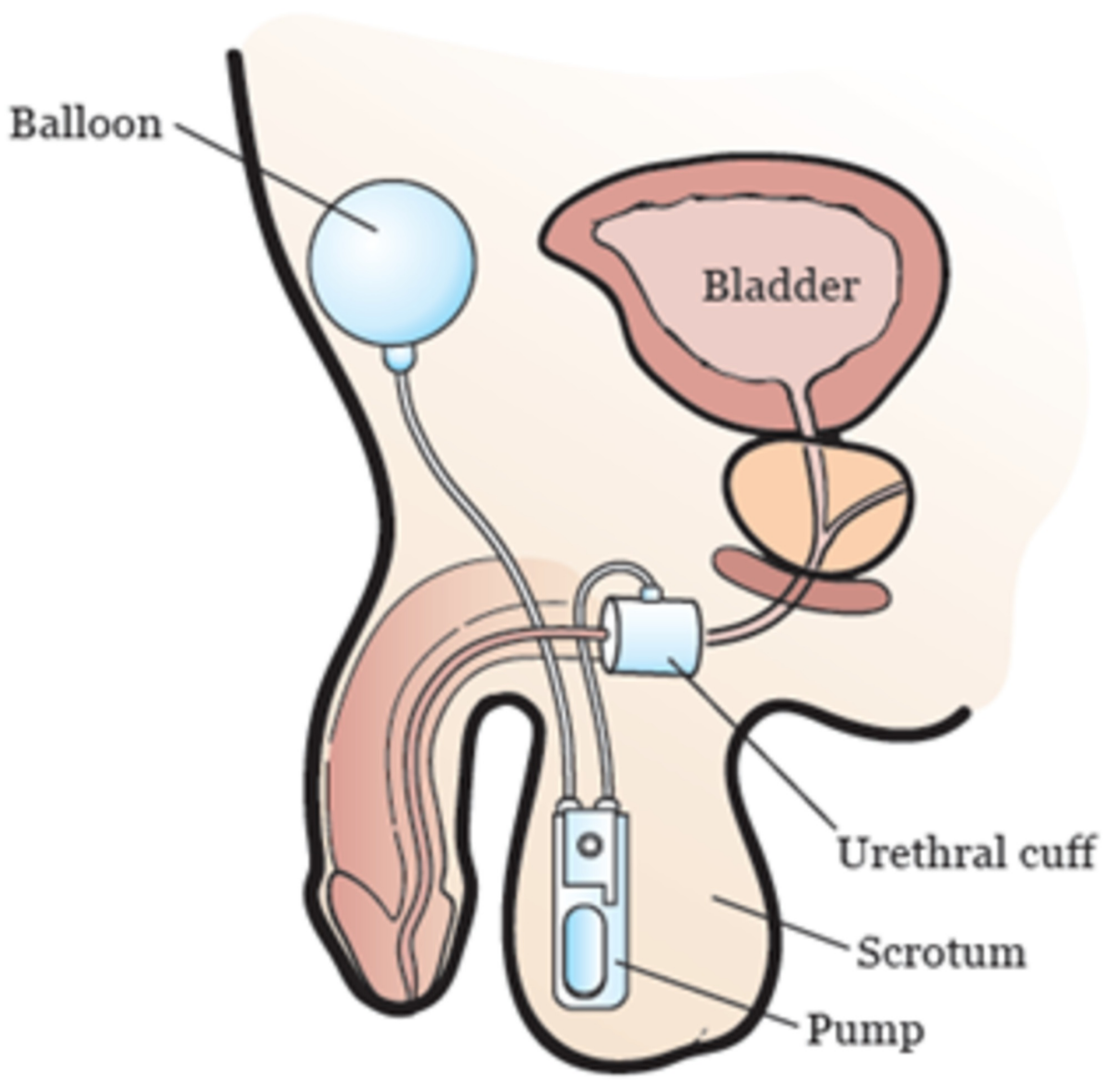
Schematic of AMS 800 artificial urinary sphincter Schematic shows the components and typical placement of the AMS 800 artificial urinary sphincter Source: MSKCC [12]

The placement of an artificial urinary sphincter is an effective form of surgical management in patients with severe post-prostatectomy urinary incontinence [9]. The AMS 800TM artificial sphincter (Boston Scientific, Marlborough, Massachusetts) is considered the industry standard for artificial urinary sphincters used to treat this form of incontinence in males [10]. The key components of these artificial sphincters include an occlusive cuff at the posterior urethra, an intraabdominal pressure-regulating balloon, and a control pump that are each connected by a fluid-filled plastic tubing (Figure 4). Artificial urinary sphincters function by manual compression of the scrotal pump when patients experience normal urgency to urinate. This thereby draws urine into the balloon, opens the urethral cuff, and allows the patient to urinate normally. After about two to three minutes, the cuff automatically closes [11].

The complications associated with the placement of an artificial urinary sphincter may include infection and mechanical malfunction (eg, fluid leakage, foley catheter trauma). Malfunction of the device can lead to urinary retention or obstructive uropathy that further predisposes patients to AKI and infection [11,13-14]. Overall, patients have demonstrated satisfaction after artificial urethral sphincter placement. Although we had an example of urinary retention; in fact, the most commonly reported complaint after device placement is leakage, with 55% of these patients reporting daily leakage of a few drops and 22% of patients reporting daily leakage of less than a teaspoon [15].

Bedside point-of-care ultrasound is useful to evaluate for possible complications associated with urinary retention, such as hydronephrosis and hydroureter, as seen in our patient. Ultrasound may also be useful to visualize artificial urinary sphincters that can help evaluate for signs of infection and the proper positioning of the device. Images of the device in our patient were obtained on scrotal and bladder ultrasound. On bladder ultrasound, a cystic mass can be visualized, which correlates with the fluid-filled balloon reservoir of these devices (Figure 3). Despite the use of artificial urinary sphincters for the treatment of urinary incontinence, there is limited ultrasound evidence of these devices. One prior study does demonstrate ultrasound images of the bladder like our patient [16]. We believe this is an important distinction to make so that this finding on bladder ultrasound is not confused with pathology [17].

Renal ultrasound should be repeated at subsequent visits to ensure the resolution of the hydronephrosis and hydroureter.

**Figure 5:**
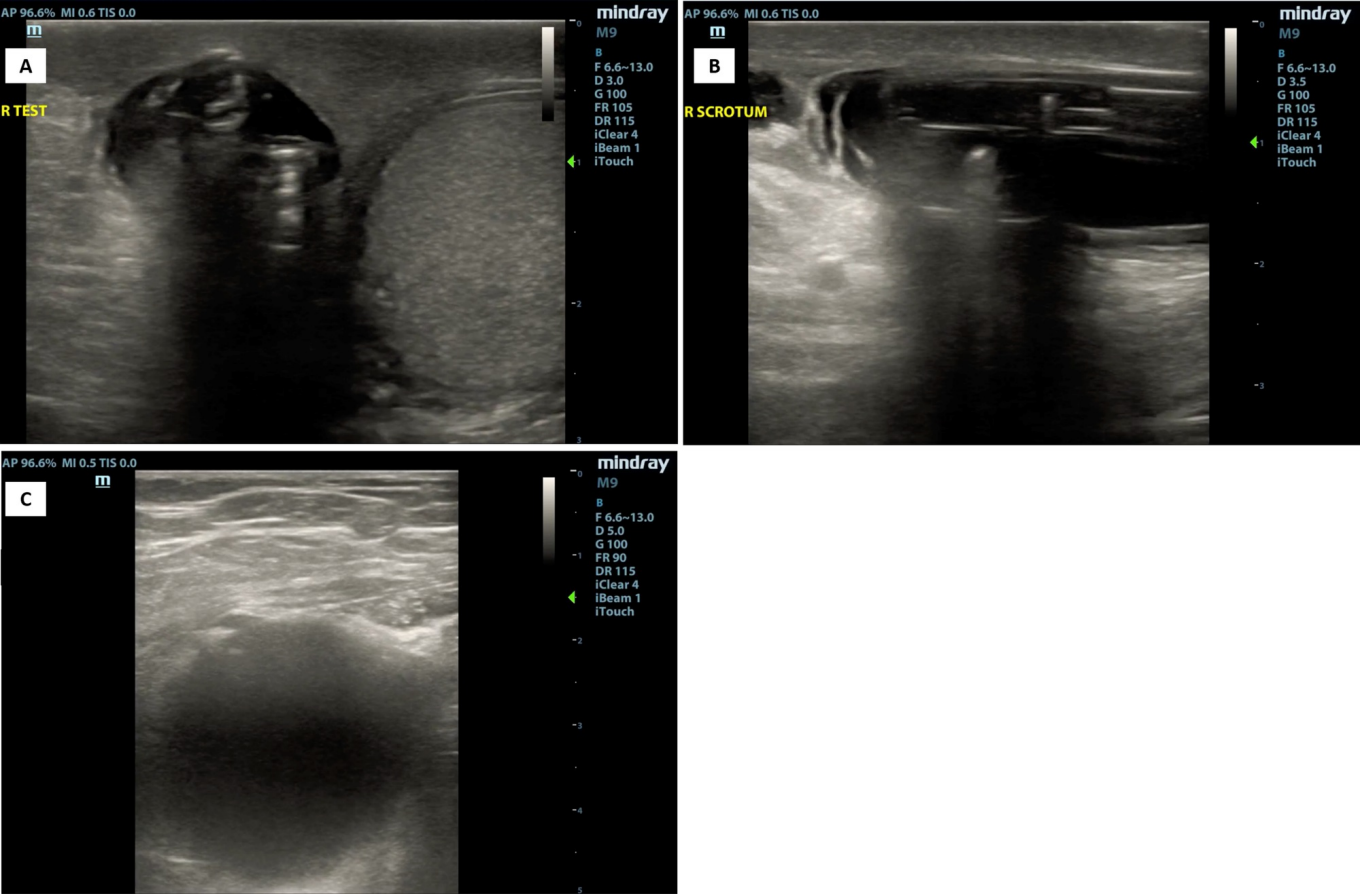
Ultrasound images of artificial urinary sphincter device Ultrasound of the right testicle demonstrates the artificial urinary sphincter location and placement seen in the long-axis view (A) and the short-axis view (B). Suprapubic view (C) also shows a visualization of the balloon reservoir.

Additional ultrasound images in our patient also demonstrate the positioning of the device without any sonographic signs of infection or inflammation (Figure 5).

After placement of an artificial urinary sphincter, urology experts have recommended to minimize urethral instrumentation since it may weaken the urethra and increase susceptibility to failure [13]. This is important for emergency physicians to be aware of to avoid straight catheterization and prevent further complications in patients with these devices.

Educational level and cognitive status have been previously studied as a possible correlation with success rates in patients with artificial urinary sphincters [18]. The patient in this case serves as an example of how important it is to provide appropriate postoperative education and ensure close-ended communication.

## Conclusions

In conclusion, we believe that emergency physicians should be aware of the artificial urinary sphincter to avoid instrumentation that can lead to a device malfunction. Patients presenting with urinary retention or a possible malfunction of the artificial urinary sphincter should be evaluated in conjunction with urology consultants. We demonstrate ultrasound evidence of the artificial urinary sphincter obtained in our patient as seen on bladder and scrotum ultrasound. We present images that may help emergency physicians who are familiar with ultrasound to ensure proper positioning of the device and to evaluate for possible signs of infection surrounding the pump in the scrotum. The bladder ultrasound images shown in our patient may be of specific interest to emergency medicine providers to avoid a possible pitfall in patients with artificial sphincters that may confuse this right-sided cystic structure with bladder pathology (eg, pathologic mass, bladder diverticulum). Although this case demonstrates a rare cause of urinary retention, the bladder ultrasound finding is consistent with the balloon of the artificial urinary sphincter. Renal ultrasound should be performed in patients with AUR to determine the presence and severity of hydronephrosis and/or hydroureter. In patients who may have communication barriers, we recommend certified interpreters to always be utilized. Patients with artificial urinary sphincters can avoid complications and ED visits by demonstrating the ability to properly activate the device.
